# Differences in clinical and biological factors between patients with PFO-related stroke and patients with PFO and no cerebral vascular events

**DOI:** 10.3389/fneur.2023.1104674

**Published:** 2023-03-14

**Authors:** Raluca Ştefania Badea, Athena Cristina Ribigan, Nicolae Grecu, Elena Terecoasǎ, Florina Anca Antochi, Sorina Bâldea Mihǎilǎ, Cristina Tiu, Bogdan Ovidiu Popescu

**Affiliations:** ^1^Neurology Department, Carol Davila University of Medicine and Pharmacy, Bucharest, Romania; ^2^Neurology Department, University Emergency Hospital of Bucharest, Bucharest, Romania; ^3^Cardiology and Cardiovascular Surgery Department, University of Medicine and Pharmacy Carol Davila, Bucharest, Romania; ^4^Cardiology and Cardiovascular Surgery Department, University and Emergency Hospital, Bucharest, Romania; ^5^Neurology Department, Colentina Clinical Hospital, Bucharest, Romania

**Keywords:** primary prevention, PFO-associated stroke, PFO (patent foramen ovale), thrombophilia, Spencer logarithmic scale

## Abstract

**Background:**

While stroke is one of the most dissected topics in neurology, the primary prevention of PFO-related stroke in young patients is still an unaddressed subject. We present a study concerning clinical, demographic, and laboratory factors associated with stroke and transient ischemic attack in patients with patent foramen ovale (PFO), as well as comparing PFO-patients with and without cerebrovascular ischemic events (CVEs).

**Patients and methods:**

Consecutive patients with PFO-associated CVEs were included in the study; control group was selected from patients with a PFO and no history of stroke. All participants underwent peripheral routine blood analyses, as well as, on treating physician's recommendations, screening for thrombophilia.

**Results:**

Ninety-five patients with CVEs and 41 controls were included. Females had a significantly lower risk of CVEs than males (*p* = 0.04). PFO size was similar between patients and controls. Patients with CVEs had more often hypertension (*n* = 33, 34.7%), *p* = 0.007. No significant differences were found between the two groups with regard to routine laboratory tests and thrombophilia status. Hypertension and gender were identified in a binomial logistic regression model as independent predictors for CVEs, but with an area under the ROC curve of 0.531, suggesting a very poor level of discrimination between the two groups.

**Discussion and conclusions:**

There is little difference between patients with PFO with and without CVEs in terms of PFO size and routine laboratory analyses. While still a controversial topic in the specialty literature, classic first-level thrombophilic mutations are not a risk factor for stroke in patients with PFO. Hypertension and male gender were identified as factors associated with a higher risk of stroke in the setting of PFO.

## 1. Introduction

Patent foramen ovale (PFO) is one of the most common congenital heart defects in adults (1) and has long been recognized as a rare cause of stroke, especially in young patients (2). However, in recent years, with the widespread implementation of minimally invasive therapeutic options for the closure of this atrial septal defect, PFO has received increased attention from both cardiologists and neurologists.

Special efforts have been made to standardize the treatment of patients with stroke and PFO and also to detect those suitable for minimally invasive PFO closure (3, 4). Since 1976, when the first case of a 17-year-old girl with PFO treated by minimally invasive methods was published (5), more than 2,800 medical papers have addressed the topics of PFO-related cerebral embolism, treatment of patients with PFO and stroke, PFO detection in patients with a history of stroke, or characterization of PFO features associated with a high risk of arterial embolism.

Nevertheless, while at this moment significant data can be found on the demographic, anatomical, and biological factors of patients with PFO and stroke, there is sparse information concerning the prevalence of these factors in patients with PFO and no cerebral ischemic events. Moreover, although significant data can be found on the selection of patients with PFO at high risk of stroke recurrence, there is little data on primary stroke prevention in persons with PFO.

We present a study whose main goal was to find specific clinical, demographic, and laboratory factors that can indicate cerebral ischemic event risk in patients with PFO.

## 2. Materials and methods

The current study was observational and aimed to identify risk factors associated with stroke/TIA in patients with PFO. A secondary goal was to characterize cerebrovascular events in patients with PFO.

### 2.1. Patient population

Participants were selected consecutively from patients hospitalized or referred to the Neurology Department of the University Emergency Hospital of Bucharest, Romania, between October 2019 and May 2022. The main inclusion criterion was a diagnosis of patent foramen ovale (PFO)-associated stroke or transient ischemic attack (TIA), defined as stroke or TIA in patients with a PFO, and no vascular or cardiac disorders predisposing to stroke, except for PFO; patients having lacunar strokes were excluded. All participants had to be between 18 and 65 years of age to be eligible for enrolment. For the purpose of brevity, the history of stroke or TIA will be referred as to a cerebrovascular event (CVE). The control group was selected from patients presenting to the same department, in the same time frame, for headache or vertigo, in whom cerebral vascular events were excluded, and who had no history of stroke/TIA, inflammatory central nervous system (CNS) disorders, or other systemic disorders with possible CNS involvement; they were invited to undergo contrast trans-cranial doppler (c-TCD) with intravenous injection of agitated saline and, in those with a positive test, a subsequent transesophageal echocardiography (TEE).

Demographical data, cardio- and cerebrovascular risk factors (hypertension, chronic kidney disease, diabetes mellitus, smoking history, and pack-years index) were recorded for both groups. For stroke patients, the involved or symptomatic cerebral vascular territory, National Institutes of Health Stroke Scale (NIHSS) score at admission and Modified Rankin Score (mRS) score at discharge were also recorded. Risk of Paradoxical Embolism (RoPE), a score developed to identify which cryptogenic strokes may be attributable to PFO, was also calculated for patients with a history of CVE.

### 2.2. Diagnosis of PFO and PFO-associated stroke

For the exclusion of other causes of ischemic stroke, all patients included in the study group had normal cervical and cerebral vascular ultrasonography, computed tomography (CT) or magnetic resonance (MR) angiography, and 24-h cardiac rhythm monitoring.

Screening for right-to-left shunt (RLS) was made for both groups using c-TCD with intravenous injection of agitated saline.

All participants in the study underwent TEE in different centers by using two types of echo machines and dedicated TEE probes (Vivid E95, GE Healthcare with 6VT-D probe and Epiq 7 Philips with X7 probe). All patients received oral local anesthesia and additional IV sedation, if needed. They were examined after transesophageal intubation from standard echocardiographic views, according to the ASE guideline for PFO assessment (6). To confirm the presence of RLS shunt iv injection of agitated saline and glucose 33% was used.

Given the differences and inconsistency of the TEE sites and methods used to diagnose the PFOs, we performed an additional standardized TEE study in our cardiology center. Data was stored and will be further analyzed in a subsequent research paper.

### 2.3. Blood analyses

All participants and controls underwent peripheral blood analysis, including leukocyte and thrombocyte count, hemoglobin level, serum triglycerides, HDL and LDL cholesterol, creatinine, fibrinogen, prothrombin time (PT), and INR levels.

On treating physician's recommendations, 115 of the patients and controls underwent screening for thrombophilia, with tests including serum homocysteine, protein C, protein S, and lupus anticoagulant levels, the activity of prothrombin and antithrombin III, and factor V Leiden, Plasminogen activator inhibitor-1 (PAI1) and Methylenetetrahydrofolate reductase (MTHFR) mutations. Since thrombophilia testing is not covered by the national insurance plan for patients without a thrombotic event, and due to the fluctuating availability of these tests in our hospital, not all patients had thrombophilia testing. The exact number of patients who were tested for prothrombotic state is depicted in the Table 1.

**Table 1:** Differences in proportions of clinical characteristics and risk factors in patients with PFO.

		**Overall (*n =* 136)**	**Cerebral ischemic events**			
			**No**	**Yes**	***p***	**Test**
**Demographical factors**						
Sex	Females	50%	63.4% (26/41)	44.2% (42/95)	**0.040**	Chi-square
	Males	50%	36.6% (15/41)	55.8% (53/95)		
Age		42.24 ± 10.92	42.66 ± 10.73	42.06 ± 11.05	0.772	Independent *t*-test
**Cardiac and cerebral risk factors**						
Hypertension		27.9%	12.2% (5/41)	34.7% (33/95)	**0.007**	Pearson Chi-Square
CKD		1.5%	0% (0/41)	2.1% (2/95)	0.349	Pearson Chi-Square
Diabetes		2.9%	0% (0/41)	4.2% (4/95)	0.315	Pearson Chi-Square
Smoker		39%	41.5% (17/41)	37.9% (36/95)	0.695	Pearson Chi-Square
Smoking index		8.77 ± 13.42	11.75 ± 15.6	7.66 ± 11.73	0.834	Mann-Whitney U
**PFO and patients related scores**						
Rank on Spencer logarithmic scale		3, IQR 1–4	3, IQR 1–4	3, IQR 1–4.25	0.426	Mann-Whitney U
RoPE			N/A	6.5, IQR 5–8		
NIHSS			N/A	3.5, IQR 1–6.75	N/A	
**Laboratory tests**						
Triglycerides (mg/dL)		86.5, IQR 56–120.7	82.5, IQR 53.5–135.2	88.5, IQR 57.7–136.5	0.077	Mann-Whitney U
HDL (mg/dL)		47, IQR 37–56	50.6 ± 11.5	45.8 ± 12.7	0.240	Independent *t*-test
LDL (mg/dL)		108, IQR 86.2–138.9	121.4, IQR 96.2–142.5	104.4, IQR 83.5–142	0.486	Mann-Whitney U
Thrombocytes (no/mm^3^)		226 500, IQR 193 750–283 250	247 500, IQR 204 000–319 625	209 000, IQR 187 250–284 000	0.186	Mann-Whitney U
Hemoglobin (mg/dL)		13.8, IQR 13–15.2	13.9 ± 1.48	14.1 ± 1.51	0.411	Independent *t*-test
Leukocytes (no/mm^3^)		7,480, IQR 5,600–10,100	6,500, IQR 5,375–9,575	7,750, IQR 5,625–11,175	0.078	Mann-Whitney U
Creatinine (mg/dL)		0.80, IQR 0.68–0.90	0.72, IQR 0.67–0.80	0.82, IQR 0.63–0.90	**0.025**	Mann-Whitney U
CKD-epi (mL/min/1.73 m^2^)		105.2, ± 17.6	105.9, ± 15.9	106.6, ± 18.9	0.190	Mann-Whitney U
Fibrinogen (mg/dL)		320.4, ± 69.9	315.7 ± 52.7	325.3 ± 82.7	0.107	Welch Independent *t*-test
**Thrombophilia status**						
PAI1		25.5% (*n =* 94)	27.6% (8/29)	23.1% (15/65)	0.760	Pearson Chi-Square
MTHFR		26.5% (*n =* 95)	39.3% (11/28)	35.8% (24/67)	0.857	Pearson Chi-Square
Factor V Leiden		1.8% (*n =* 113)	0% (0/29)	2.4% (2/84)	1	Fisher's Exact test
Antithrombin III		2.6% (*n =* 114)	0% (0/29)	3.5% (3/85)	0.569	Fisher's Exact test
Protein C deficiency		3.5% (*n =* 115)	3.5% (1/29)	3.5% (3/86)	1	Fisher's Exact test
Protein S deficiency		5.2% (*n =* 115)	0% (0/29)	7% (6/86)	0.335	Fisher's Exact test
High homocysteine serum level		22.6% (*n =* 115)	17.2% (5/29)	24.4% (21/86)	0.424	Pearson Chi-Square
Lupus anticoagulant		3.5% (*n =* 115)	3.4% (1/29)	3.5% (3/86)	1	Fisher's Exact test
Prothrombin mutation		0	0	0	-	-

The bold values are the values with statistical significance (where statistical significance was considered at a *p* value less than 0.05).

### 2.4. Transcranial doppler and intravenous injection of agitated saline

c-TCD was performed in all participants and controls using a Digital DWL^®^ TCD device with DWL^®^ routine software (Doppler-Box™). All examinations were made by the same examiner using a standard protocol, which consisted of insonation of both middle cerebral arteries (MCAs) at 55 mm using 2 MHz PW monitoring probes mounted on a DiaMon^®^ headset, DWL USA, Inc. A 23-gauge cannula was inserted in one of the antecubital veins and one milliliter (mL) of blood was extracted from the patient and mixed with 1 mL of air and 8 mL of 0.9% saline solution. The compound was mixed ten times using two syringes and a three-way stopcock and injected into the intravenous cannula. The patient was asked to perform two Valsalva maneuvers. The total number of microbubbles that passed through both MCAs was counted. The grade of the right-to-left intracardiac shunt was estimated using Spencer's logarithmic scale (SLS) (7).

### 2.5. Statistical analysis

For continuous variables, the Shapiro-Wilk test was performed to determine the normality of the data distribution. Continuous variables with normal distribution are presented as mean ± standard deviation, while those with non-normal distribution are presented as median (interquartile range). Categorical variables are reported as the rate (percentage). For comparing differences in demographical, clinical, or laboratory characteristics between PFO patients with cerebral ischemic events and controls, the independent *t*-test was conducted for continuous variables, and the χ2 or Fisher's exact test was used for categorical variables. Outliers were kept in the analysis as they depicted actual clinical scenarios. The homogeneity of variances was assessed by Levene's test of homogeneity of variances. Mann-Whitney U test and Kruskal-Wallis H tests were run to determine if there were differences between two and three or more groups of non-normally distributed variables (8). Linearity of the continuous variables with respect to the logit of the dependent variables was assessed *via* the Box-Tidwell (1962) procedure. To determine the relationships between variables, the Pearson correlation test was performed. In the case of non-linearity, Spearman's rank-order correlation test was run. Binomial logistic regression was run to analyze the effects of clinical variables on the likelihood of a cerebral ischemic event. Variables were selected based on the univariate regression analysis results and introduced in the model using the one-step enter method. Crude and adjusted odds ratios (OR) and their respective 95% confidence intervals (CI) were calculated and reported.

The study was approved by the Board of Ethics of the University Emergency Hospital of Bucharest. All patients signed a consent stating their non-opposition to the use of medical data, including its publication. The research was performed in accordance with the Declaration of Helsinki and GDPR standards.

## 3. Results

### 3.1. Patient population

Ninety-five patients fulfilling the inclusion criterion were identified (55.8%, *n* = 53 males, mean age = 42.06 ± 11.05); of those, 73 had a stroke (58.9%, *n* = 43 males, mean age = 42.6 ± 10.8) and 22 had a TIA (45.5%, *n* = 10 males, mean age = 40.1 ± 11.8). For controls, 189 candidates were initially considered; after undergoing c-TCD, patients without RLS were excluded, and 41 participants were finally included (36.6%, *n* = 15 males, mean age = 42.66 ± 10.73).

### 3.2. Demographical factors

Mean age for CVE patients (42.06 ± 11.05) and controls (42.66 ± 10.73) was not statistically significantly different t(134) = 0.291, *p* = 0.772. Females had a significantly lower risk of CVEs than males (*p* = 0.04), the OR of CVE in females vs. males being 0.792 (95% CI, 0.632–0.993).

### 3.3. PFO-associated stroke group characterization

**Figure 1:**
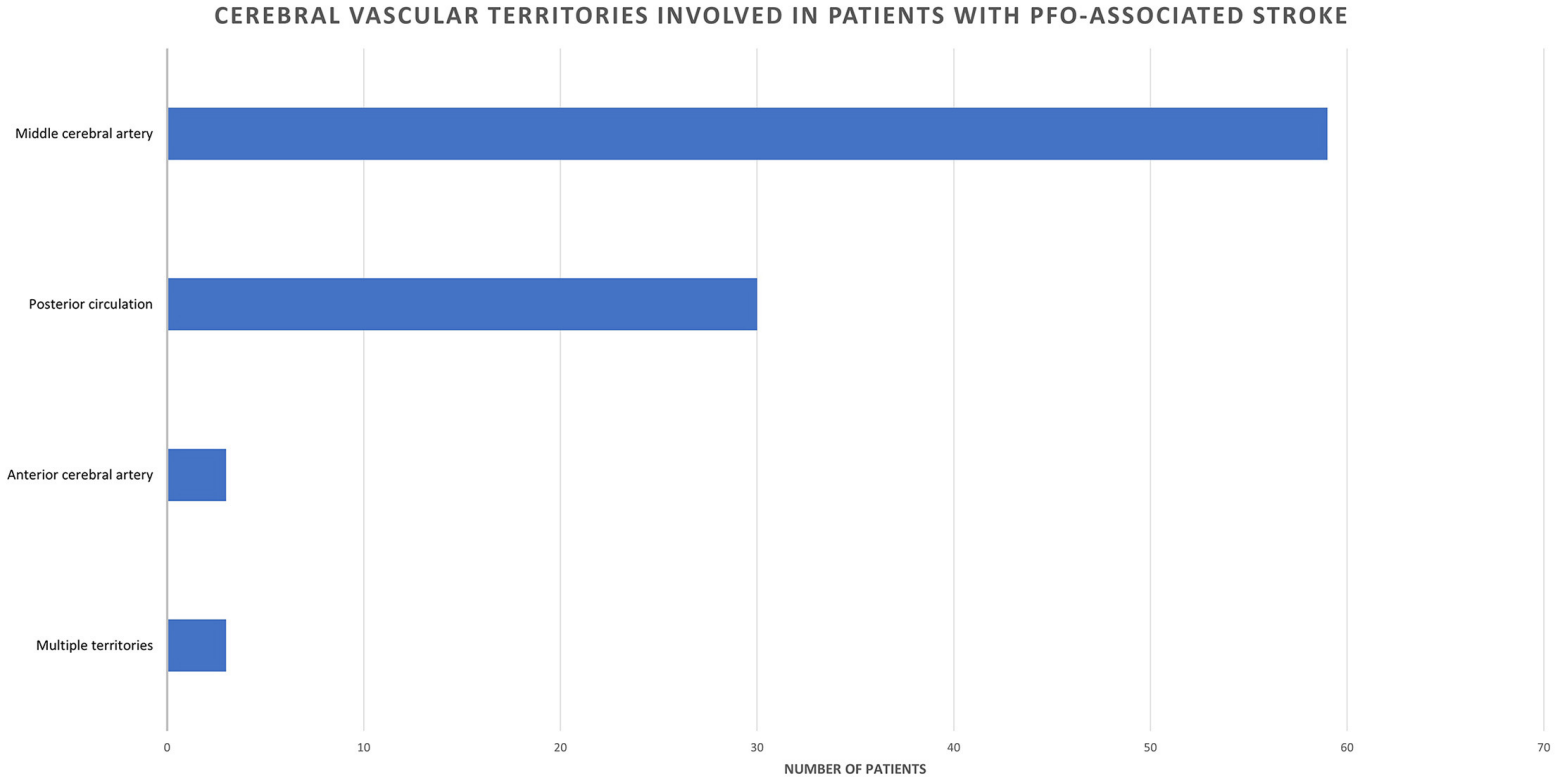
Cerebral vascular territories involved in patients with PFO-associated stroke.

The most often involved cerebral vascular territory in CVE patients was the middle cerebral artery (MCA) (62.1%, *n* = 59), followed by the posterior circulation (31.5%, *n* = 30), and the anterior cerebral artery (ACA) territory (3.2%, *n* = 3); in 3.2% (*n* = 3) of patients, stroke occurred in multiple cerebral vascular territories (Figure 1).

Median NIHSS score at admission of patients having a PFO-associated stroke was 3.5 (IQR = 6). A Spearman's rank-order correlation was run to assess the relationship between PFO shunt size and stroke severity, as assessed by the NIHSS score. There was no statistically significant correlation between PFO shunt size and stroke severity r_s_(71) = 0.165, *p* = 0.186.

**Figure 2:**
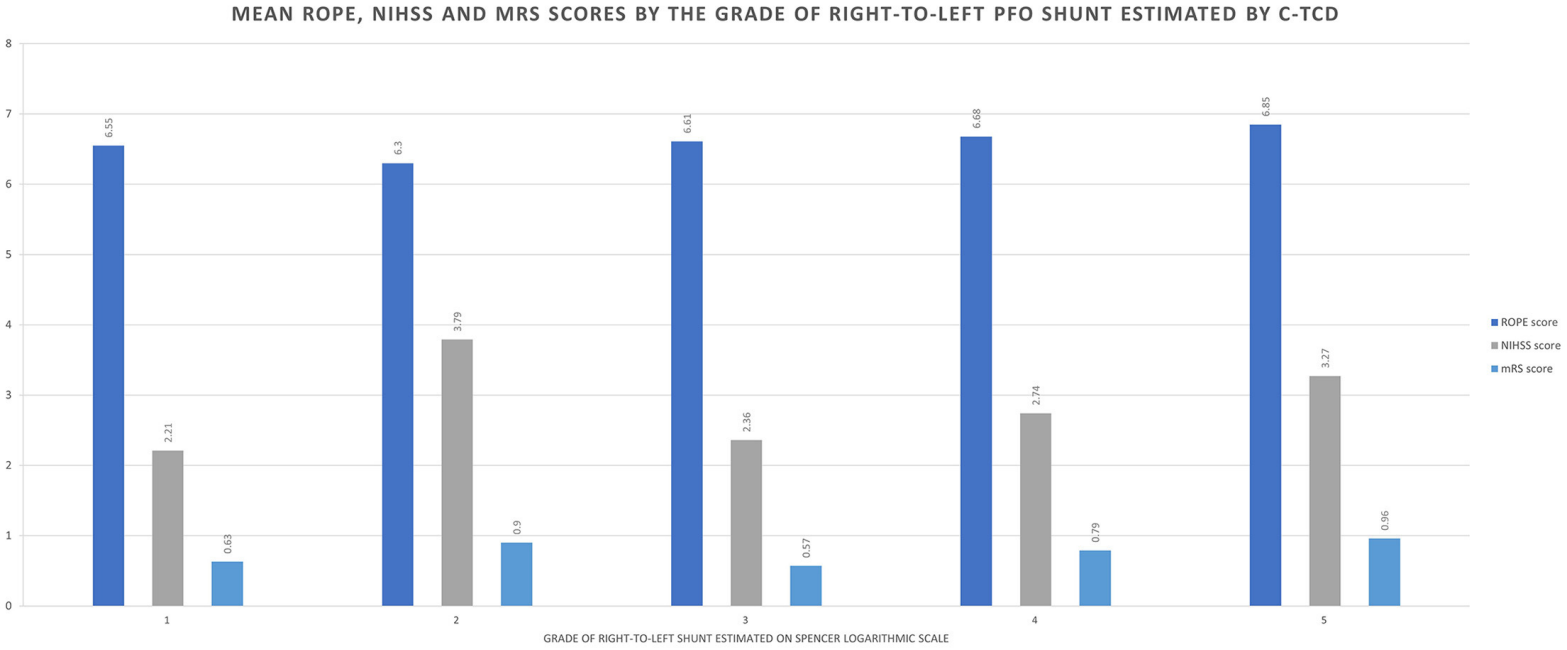
Characteristics of PFO-associated stroke depending on PFO size estimated on Spencer's logarithmic scale (1–5).

At discharge, most of the stroke patients had mRS scores between 0–2 (*n* = 62, 84.9%), the remaining having mRS score of 3 (*n* = 10, 13.7%), and respectively of 4 (*n* = 1, 1.4%). Characteristics of PFO-associated stroke depending on PFO shunt size estimated on Spencer's logarithmic scale are depicted in Figure 2. The complete characterization of the two groups is further detailed in the *Group comparison* section.

### 3.4. Differences between PFO patients with and without a stroke—Group comparison

#### 3.4.1. PFO shunt size (Rank on Spencer's logarithmic scale)

The grade of the PFOs right-to left shunt as assessed by c-TCD using the SLS did not differ between CVE patients and controls, median score on the SLS being 3 in both groups, [χ2(2) =0.633, *p* = 0.426].

#### 3.4.2. Cardiac and cerebral risk factors

When analyzing the differences in laboratory tests and cardiac- and cerebral risk factors between the two groups, patients with CVEs had statistically significant more often hypertension (*n* = 33, 34.7%) than controls (*n* = 5, 12.2%), *p* = 0.007. It was also noticed that patients with a history of cerebral ischemic events had more frequent chronic kidney disease (CKD) and diabetes mellitus compared to controls but were less often smokers and had a lower smoking index; however, this did not reach statistical significance.

#### 3.4.3. Laboratory tests

No significant differences were found between the two groups with regard to routine laboratory tests. While CVE patients had significantly higher values of serum creatinine (0.81 mg/dL, IQR = 0.24) compared to controls (0.70, IQR = 0.15), *U* = 1,696, *z* = 2.246, *p* = 0.025, testing for differences in the glomerular filtration rate, as estimated by CKD-EPI, found no differences between the two groups. Patients with a history of cerebral ischemic events had higher triglycerides, LDL cholesterol fraction, hemoglobin, leucocytes, and fibrinogen levels, while controls had higher HDL cholesterol fraction values and higher thrombocyte counts, but none of these findings reached statistical significance. Details concerning the laboratory tests' differences between the two groups can be found in Table 1.

#### 3.4.4. Thrombophilia status

Of all patients included in the study, 115 underwent screening for classical thrombophilia tests. The exact number of patients having each one of the tests is depicted in the Table 1. No significant differences were found between PFO patients with and without a history of cerebral ischemic events concerning thrombophilia status. When compared to controls, patients with CVEs had more frequent mutations of the factor V Leiden, abnormal activity of antithrombin III, protein C and S deficiency, and abnormal lupus anticoagulant and homocysteine levels, but no statistical significance was reached. On the other hand, PAI1 and MTHFR mutations were more frequently observed in patients without a history of a cerebral ischemic event, again, without reaching statistical significance. In our population, no patients had prothrombin mutation. Results are depicted in Table 1.

### 3.5. Factors predicting stroke in PFO patients

Two independent predictors for CVEs were identified in a binomial logistic regression model with 69.9% classification accuracy: hypertension (OR 0.27, CI 0.02–0.77, *p* = 0.014) and gender (OR 0.48, CI 0.22–1.05, *p* = 0.06). Nevertheless, the area under the ROC curve was 0.531, which, according to Hosmer et al. (9), suggests a very poor level of discrimination between the two groups.

## 4. Discussion

Our data suggest that sex is a determinant of risk of stroke in patients with PFO, males having a significantly higher risk of CVEs compared to female patients. This may be to the fact that population included in the study was predominantly young, and male patients have an overall risk of stroke higher than female patients aged <65 (10). Moreover, similar sex ratios were also reported in studies describing populations with PFO-related strokes, Mazzucco et al. reporting a 55.2% frequency of male gender in the PFO-related stroke population (11), as well as in the RESPECT trial, in which 54.7% of patients with ischemic stroke and evidence of PFO were males (12).

Literature data regarding vascular territory involvement in PFO-related stroke vary, with studies focusing either on imaging characteristics helping to differentiate these cases from other causes of stroke (13–17), or specifically on PFO (18, 19). Our data is in accordance with the results of Kim et al. (15) and of Nam et al. (18) in that carotid artery territory, and specifically the MCA, is most commonly affected in PFO-related stroke. On the other hand, the majority of the other works report either posterior circulation or multiple territories as being more commonly involved. We cannot ascribe this difference to any specific factor and believe it to be in line with reports regarding general stroke vascular distribution.

In our population, PFO-related stroke severity as measured by the NIHSS was mild, with patients having a median NIHS score of 3.5. This is in accordance to the results of a study presented by Kaito Abe et al., which found a mean NIHSS score of 2.2 ± 2.8 in patients with PFO-related stroke (20). There is less data regarding the functional outcome in patients with PFO related strokes, but one recent paper reporting on stroke in patients with no established vascular risk factors noted that this group of patients, in which PFO-related stroke was included, has a worse outcome at 12 months (21); however, individual data on PFO-related stroke was not provided. In our cohort, stroke severity was mild, and functional outcome was generally good.

The number of microemboli detected through TCD, and especially during the Valsalva maneuver, has been shown to correlate to both a large PFO size and a high-risk morphology of PFO, as assessed through TEE (22, 23). While most of the current literature data are in agreement that multiple PFO anatomical characteristics are related to a high-risk of embolization, when it comes to PFO size *per se*, reports are somewhat conflicting. This may be partly due to the various methods used in PFO detection, as well as to the endpoints of each trial. In a cohort of PFO-related stroke patients described by Sadrameli et al. (24), a high-grade shunt as measured by TCD was only encountered in a minority of stroke patients, and an analysis of pooled data from several studies by Turc et al. (25) identified the presence of atrial septal aneurysm as a more important predictor of recurrent stroke compared to shunt size. In our cohort, PFO shunt size, as assessed by TCD, was average in both CVE and non-CVE groups. This might indicate that other factors pertaining to morphology of the PFO accounted for the CVEs in the respective subgroup.

Patients with a history of CVE had significantly more often hypertension than stroke-free patients. All enrolled patients were young patients, with no atheromatosis and all those with lacunar strokes were excluded. Thus, the role of hypertension in stroke occurrence in this group of patients is unclear.

Changes of the endothelium of blood vessels in the incipient stages of hypertension might be one of the first steps in thrombogenesis. In an editorial published in 2020, Lip and Blann describe the possible imbalance between the coagulation and fibrinolytic pathways present in hypertensive patients (26). Moreover, in recent years, there have been reports supporting the role of hypertension-induced circulating endothelial and platelet microparticles in thrombi formation (27). A study published in 2020 by Silambanan et al. showed that patients with hypertension, even in incipient phases, have high blood concentrations of endothelial microparticles (28), markers that can initiate or promote vascular dysfunction and thrombosis (27).

Therefore, we believe that hypertension may have an important role as an independent factor in the pathogenesis of stroke in patients with PFO, and caution should be exercised when disregarding PFO as a cause for stroke in patients with hypertension or lower ROPE scores.

The role of thrombophilia status in the occurrence of stroke in patients with PFO is still a controversial topic in the literature. Indeed, several studies reported increased prevalence of classical hypercoagulable states in patients with PFO and CVE. Pezzini et al. found a significantly higher prevalence of either prothrombin or Factor V Leiden mutation among 35 stroke patients with PFO, compared with 149 controls (29). Similar results are reported by Karttunen et al. (30). However, in both studies, controls were not tested for PFO and were not assessed by imaging to exclude silent cerebral infarcts. Nevertheless, in our study, none of the patients had prothrombin mutation. More conclusive studies are addressing the benefit of PFO closure in patients with thrombophilia for secondary prevention of CVEs. In a review and meta-analysis published in 2019, Hviid V. B. and colleagues reported that a positive thrombophilia status could be considered an independent factor for the recurrence of stroke in PFO patients (31). Nevertheless, the antithrombotic treatment was not consistent between patients who benefited from PFO closure and those kept only on medical treatment. In most of the studies, patients who benefited by PFO closure and thrombophilia were more often treated with anticoagulants or dual antiplatelet therapy after the procedure, a factor that may have contributed to the lower recurrence of CVE in treated patients. In a cohort study which included only stroke-free patients with PFO and a previously known hypercoagulable state, Buber et al. also reported that PFO closure in patients with thrombophilia resulted in a lower risk of CVE, independent of the type of antithrombotic medication (32). In accordance with these findings, the most recent published guideline by the Society for Cardiovascular Angiography & Interventions recommends against the closure of PFO in patients with thrombophilia and without previous stroke, and closing the PFO in patients with prior stroke and thrombophilia. Nevertheless, both recommendations are conditional and considered with very low certainty of evidence (33).

In our study, the prevalence of different classical first-level thrombophilic mutations was similar in both groups, and for most of the thrombophilia, it was somehow similar to the prevalence in the general population (34–37). One exception was the prevalence of protein C deficiency, which in previous reports was significantly lower (0.14–0.5%) (38, 39) than in our studied population (3.5%). Though epidemiological data on thrombophilia prevalence is not available for Romania, this difference could be due to the geographical variations of different mutations. Considering the aforementioned findings, one may be inclined to believe that the presence of classical first-level thrombophilia mutations has no role in the occurrence of a cerebral ischemic event in patients with PFO, and is more likely to be an incidental finding.

Nevertheless, more subtle thrombophilic changes have been described in patients with migraine and intracardiac right-to-left shunt. In a recent paper, Trabattoni et al. reported that when compared with healthy subjects, patients with migraine and PFO had an increased procoagulant status secondary to a higher platelet expression of tissue factor and annexin V binding to phosphatidylserine, as well as an increased oxidative stress status, and increased platelet activation (40). These findings may suggest that other procoagulant pathological mechanisms may be involved in CVE occurrence in patients with PFO, leaving the door open for newer other markers.

As expected, even though with a moderate sensitivity, a predictive model consisting only of hypertension and gender had no specificity in predicting stroke occurrence in patients with a PFO. The authors believe that adding specific morphological characteristics of the PFO to the model would improve both sensitivity and specificity of the model. This will be addressed in future reports.

The current study provides not only a thorough description of clinical characteristics and laboratory tests results of patients with PFO-related stroke, but also assesses the differences between patients with PFO and stroke and those without one. Our study showed that there is little difference between patients with PFO and a history of stroke/TIA and those without a cerebral vascular event. Though much attention is now given to specific factors that can be associated with a higher risk of recurrence of stroke in patients with PFO, our study demonstrates that risk factors that are usually associated with cardiac and cerebral ischemic events (tobacco usage, dyslipidemia, diabetes) have no role in PFO-associated stroke. Moreover, thrombophilia and intracardiac shunt size, though highly controversial subjects, have no role in stroke occurrence in patients with PFO. Interestingly, the only two factors associated with a higher risk of stroke in our cohort were hypertension and male gender. The findings of this study represent a cornerstone in the possible primary prevention of stroke in young patients with PFO, and in detangling the unknown factors that contribute to cerebral ischemic events in the large PFO population.

These findings, along with the paucity of data concerning primary prevention of stroke in patients with PFO are emphasizing the need for further large sample sized studies.

## 5. Strengths and limitations

Our study has a major strength. This is one of the first studies to describe the differences between patients with PFO with and without a first ischemic event, thus marking the risk factors that might be involved in the pathogenesis of an ischemic stroke in patients with PFO. However, our study has several limitations. Firstly, the study group was significantly larger than the control group, and the control group comprises a higher ratio of females than the study group. In general, young stroke patients are more frequently males; however, in this particular study, the significance of the statistical association may have been overestimated by choosing a female-predominant control group. A headache-free PFO carriers group should be the ideal control group. While the authors are aware of these major limitations, we believe it reflects the real-life data and the profile of an Emergency Hospital. Another limitation is the absence of data concerning morphological aspects of the PFO, which may have had an important role in differentiating patients with PFO and stroke of those without CVE; while this data is not available at the moment, it will be presented in a future report. Additionally, thrombophilia tests were not available for all patients included in the study and were performed only once, and in the case of patients with ischemic stroke, these results may have been influenced by the acute inflammatory changes that occur in patients with acute stroke. Finally, another limitation is that being a single-center study, the results may limit the generalizability of the study.

## Data availability statement

The raw data supporting the conclusions of this article will be made available by the authors, without undue reservation.

## Ethics statement

The studies involving human participants were reviewed and approved by Board of Ethics of the University Emergency Hospital of Bucharest. The patients/participants provided their written informed consent to participate in this study.

## Author contributions

RB, FA, AR, and SB were involved in protocol development and patient recruitment. RB and ET were involved in the data analysis. RB and NG wrote the first draft of the manuscript. CT and BP reviewed and edited the manuscript and approved the definitive version of the manuscript. RB edited and submitted the final paper. All authors contributed to the article and approved the submitted version.
